# Radon—The Element of Risk. The Impact of Radon Exposure on Human Health

**DOI:** 10.3390/toxics8040120

**Published:** 2020-12-14

**Authors:** Anna Grzywa-Celińska, Adam Krusiński, Jadwiga Mazur, Katarzyna Szewczyk, Krzysztof Kozak

**Affiliations:** 1 Chair and Department of Pneumonology, Oncology and Allergology, Medical University of Lublin, 20-090 Lublin, Poland; adak93@gmail.com; 2 Institute of Nuclear Physics, Polish Academy of Sciences, 31-342 Krakow, Poland; jadwiga.mazur@ifj.edu.pl (J.M.); krzysztof.kozak@ifj.edu.pl (K.K.); 3 Chair and Department of Pharmaceutical Botany, Medical University of Lublin, 20-093 Lublin, Poland; k.szewczyk@umlub.pl

**Keywords:** radon, radiation, exposure, lung cancer

## Abstract

Lung cancer is a heterogeneous group of diseases with multifactorial aetiology. Smoking has been undeniably recognized as the main aetiological factor in lung cancer, but it should be emphasized that it is not the only factor. It is worth noting that a number of nonsmokers also develop this disease. Radon exposure is the second greatest risk factor for lung cancer among smokers—after smoking—and the first one for nonsmokers. The knowledge about this element amongst specialist oncologists and pulmonologists seems to be very superficial. We discuss the impact of radon on human health, with particular emphasis on respiratory diseases, including lung cancer. A better understanding of the problem will increase the chance of reducing the impact of radon exposure on public health and may contribute to more effective prevention of a number of lung diseases.

## 1. Introduction

While many known factors contribute to carcinogenesis, the tumorigenic process is usually the result of a combination of genetic and environmental factors. The effects of some of them are modifiable, while the exposure to others is barely manageable. The harmful effects of tobacco smoke, environmental pollution, poor diet, abnormal weight and low physical activity are commonly known. Other factors are yet to be thoroughly recognized, and therefore the measures to eliminate them are less common.

The purpose of our work is to draw attention to environmental exposure to radon as an aetiological factor in respiratory diseases, including lung cancer, especially in nonsmokers.

This review answers several important questions about the impact of radon exposure on human health. It brings up a topic of partially modifiable risk factor of lung cancer, exposure to radon, which is the second largest risk factor of lung cancer for smokers and first in nonsmokers.

## 2. Chemical Properties of Radon

Radon is an odorless and colorless noble gas. There are four known natural isotopes including the most stable and also the isotope most significant to health, Rn-222. Its radioactivity is a characteristic feature of this element. Radon comes from the uranium decay chain (U-238), arising directly from radium decay (Ra-226). Radon decays into further radioactive elements, up to a stable lead isotope Pb-206. Its half-life is only 3.82 days. It is worth emphasizing that among radon decay products are also alpha and beta radioactive isotopes. When combined with dust and aerosol particles present in the air, they can be aspired and deposited in the human respiratory system where they decay and become a source of significant radiological exposure [1,2]. Radon derivatives do not deposit evenly in the respiratory system: the depth of penetration of a particle depends on its size.

Alpha (α) particles are made up of two protons and two neutrons, so they have the structure of helium nuclei. They ionize and damage the DNA found in the cells of living organisms. This radiation has a very small range and is blocked by human skin, but it can be a carcinogen if it enters the body, e.g., the respiratory tract as radon can be easily inhaled. Beta (β) and gamma (γ) radiation penetrate deeper into the body and can also cause damage to genetic material, leading to the development of a malignant tumor [3,4].

Radon is a noble gas and is generally chemically inactive. It is quite mobile and can migrate both in the Earth’s crust and in the air. Radon concentrations are measured in becquerels per cubic meter of air. One becquerel (1 Bq) is one disintegration per second, which is a very small unit, so units such as the curie (Ci) are used; 1 Ci is 37 GBq. For example, the activity of one gram of Rn-222 equals 1.538 × 10^5^ Ci [5]. The dose of ionizing radiation absorbed by the body is expressed in Gy (gray) units and one gray is the energy of one joule absorbed by one kilogram of body weight (J/kg). The assessment of the actual effect of radiation on the human body requires the use of doses equivalent which account for the type of organ and the type of radiation. The lungs, stomach and bone marrow have a weight ratio of 0.12, and the skin ratio is 0.01. The multiplier for α radiation is 20, and that of electrons and photons is only 1 [6]. The unit of dose equivalent is the sievert (Sv), which, like the gray, is a joule of absorbed energy per kilogram of body weight. A 1 J/kg dose poses a threat to human health [7].

## 3. Natural Sources of Radon

Radon is responsible for approximately 40% of radiation to which humans are exposed [4] and is the main source of natural radiation [8,9]. It comes primarily from the soil, building materials, water and natural gas [10,11,12].

The source of radon in the air is the Earth’s crust, which contains the direct predecessor of radon in the radioactive chain, i.e., radium (Ra-226), whose decay leads to the formation of radon (Rn-222). Radon from the decay of radium in geological formations is transported to the Earth’s surface as a result of diffusion and convection. The amount of radon exhalation (extraction) depends on the location (soil type, soil geology) and atmospheric conditions (pressure, wind strength and direction, humidity, snow cover, etc.). The extent of exhalation is also closely correlated with the occurrence of tectonic faults. Such faults are an excellent path for radon migration even from deep geological layers [13,14,15].

Granite soils show high uranium content, while the content in sedimentary rocks is low. Active seismic zones, tectonic movement areas, volcanic zones and geothermal fields are significant sources of radon.

In Poland, radon occurs primarily in the Sudetes and Sudeten Foothills, where granitoid massifs and metamorphic rocks with increased uranium and thorium contents are found. The second area of the high occurrence of radon is Upper Silesia, in particular its coal-mining region. In addition to granites, large amounts of radium may contain shale and phosphate rock.

Sudeten rocks, mainly igneous, contain large amounts of uranium and radium even at low depths, reaching up to 200 m. They have a mosaic geological structure, with a lot of cracks, brittle rocks and tectonic dislocations, which make it easy for the gas to move upwards. This movement is also facilitated by flowing groundwater and carbon dioxide [16,17].

Radon is found in groundwater, which is associated with its flow through rocks rich in this element and showing a high emanation coefficient. In the Sudetes, the most radon is found in low-mineralized waters with a short groundwater flow period of several years. The concentration of the radioactive element reaches up to 2000 Bq/L [18,19]. Similarly, the content of radon in natural gas results from the fact that they occur in soil rich in elements of the uranium chain.

The concentration of radon in atmospheric air in the open is usually low. In Poland, it varies from a few to several dozen Bq/m^3^. In houses, this concentration can be much higher: from several dozen to several thousand Bq/m^3^ [20].

### Geographic Distribution of Radon

Data covering the 27 European countries [21] show that radon is responsible for around 8% of lung cancer deaths. In the UK, the proportion is estimated at 3.3% (0.2% of all deaths). The US Environmental Protection Agency (EPA) estimates that 1 in 15 homes in the U.S. (about seven million) have elevated radon levels [22].

The 2009 WHO report shows the arithmetic mean of indoor radon concentrations in many countries, e.g., 140 Bq/m^3^ in the Czech Republic and Mexico, 49 Bq/m^3^ in Poland, 46 Bq/m^3^ in the USA, 28 Bq/m^3^ in Canada, 20 Bq/m^3^ in the UK, 16 Bq/m^3^ in Japan and 11 Bq/m^3^ in Australia. The global average was 39 Bq/m^3^ [23].

Since 2006, research has been carried out in Europe to create an atlas with average indoor radon concentrations in rooms at ground level. In 2014, these studies were already conducted in 24 countries. The distribution of results correlates with the geological conditioning of the site. High radon concentrations were observed in the granitic areas, for example, the Bohemian Massif, the Iberian peninsula, the Massif Central, the Baltic shield, Corsica, Cornwall, the Vosges mountains, in the Central Alps, the Swiss Jura, the Dinarides, north Estonia and in volcanic parts of Italy. The arithmetic mean for Europe was 98 Bq/m^3^, and the median was 63 Bq/m^3^, but there were large differences between different countries—for example, in the Czech Republic over 90% of the country’s area had an average indoor radon concentration above 100 Bq/m^3^, while in Lithuania this percentage did not exceed 10%. For the entire examined area of Europe, the percentage of the studied area with indoor radon concentration exceeding 100 Bq/m^3^ was approximately 25, and for concentrations over 300 Bq/m^3^, about 5% [24].

Research conducted in the USA shows large differences in radon levels, with the highest concentrations in the Northeast and Upper Midwest. The highest radon concentrations were recorded in Pennsylvania (844 Bq/m^3^), and the lowest in Florida, Louisiana and California (11.1 Bq/m^3^) [25].

## 4. Radon in Buildings

Radon escaping from the Earth’s crust and into atmospheric air can penetrate buildings. The share of radon in the air inside a statistically representative building, assuming full air exchange every hour, is as follows: the subsoil accounts for nearly 80% of the radon source, the second source is in building materials responsible for 12% of radon, and the third is atmospheric air—9.3%. Water and natural gas together account for less than 1% [11,12].

**Figure 1:**
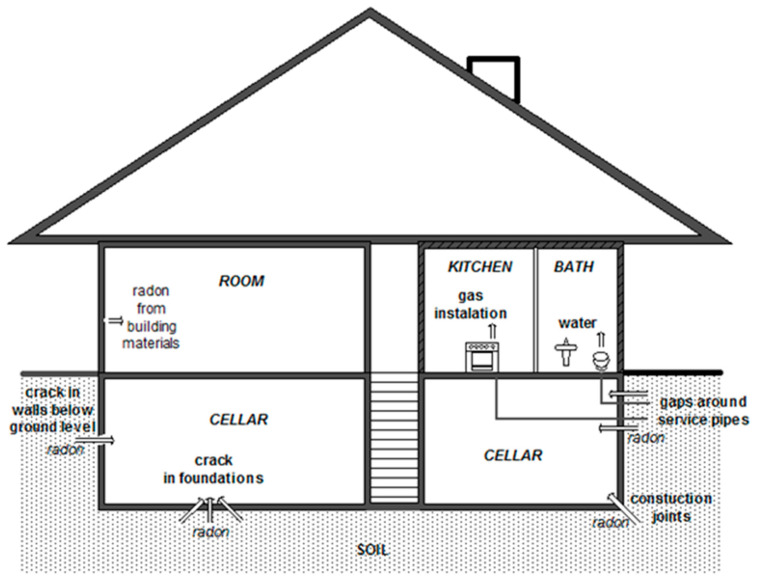
Radon pathways into buildings.

Radon is much heavier than air (7.6 times) and should remain in the basement layer but the foundation of the building changes this situation. Building a house requires “penetrating” the soil surface and reaching deeper layers, where radon concentrations are much higher due to the content of ^226^Ra radium isotope. The basic mechanism for the entry of this gas into houses is always the pressure difference between the inside and the outside—the pressure inside is a few pascals lower than outside the building. This phenomenon is caused by devices, such as those for sewage or ventilation, which work in a house and “pump out” the air. Another reason is the fact that the house has a higher temperature than outside. Warmer air is lighter and thinner, it produces less pressure and causes radon to escape from the soil and rise. Radon is also suctioned from the lower rooms and building walls (Figure 1). The main ways in which radon penetrates inside buildings are leaks in the building, such as cracks and crevices of the concrete screed, structural gaps and cracks in the building, cracks in the walls which have direct contact with the ground, cracks in the walls and leaks around sewage pipes.

Another source of radon in houses is its escape from walls and ceilings made of materials which always contain some amount of radium. Generally, it can be stated that radon from the ground dominates on the lower floors of the building, and the higher the floor, the greater the share of radon coming from building materials used in the construction. Higher levels of radioactivity are found in industrial raw materials: fly ash, slag, phosphogypsum and concrete [26].

Another source of radon is water because radon is released from it when domestic sanitary facilities (e.g., showers, baths) are used.

The concentration of radon inside buildings shows high daily and seasonal variability. In autumn and winter, when temperatures are low outside, doors and windows remain closed most of the time and radon concentrations in rooms reach much higher values.

Research carried out in Switzerland by Kropat and coauthors identified a number of factors affecting indoor radon concentrations (IRC). Less radon was found in newer buildings. It was noted, however, that radon concentrations remained at a higher level in single-family houses as compared to flats [27]. In a single-family house, the transport of radioactive radon from the ground can reach 15 kBq/h. This is mainly through advection, which depends on the difference between the outside and inside temperatures, and the movement of air over radon-rich soil [28]. Yu et al. investigated typical Hong-Kong buildings and found that radon release from the concrete walls of older buildings was smaller [29]. However, it is worth noting that building materials are a secondary source of radiation. Research in the UK has revealed a strong correlation between radon concentration and socioeconomic status. It turned out that lower levels of radon are detected in the houses of less affluent people. Factors considered to be the causes include the inferior insulation and lower temperature prevailing in such houses. The exposure to radiation generated by radon is lower but less affluent people smoke statistically more cigarettes, which is an indisputable risk factor for lung cancer [30].

The content of radon in the interior of the house is also affected by the exchange of air with the external environment. This occurs by diffusion and convection - the latter, like advection, depends on temperature and increases with its gradient [28,31]. Airing the apartment facilitates those processes. It is estimated that a tilted window can reduce radon levels in an interior by up to 70% [32].

Building materials are a less important source of radiation than the soil on which the building is erected. They contain radon, which is naturally produced, much like other radioactive elements of the uranium chain, thorium chain and the K-40 potassium isotope. In addition to α radiation, building materials also emit γ radiation. Some of this radiation also enters buildings from space. In practice, building materials are assessed with the use of two activity indicators, f1 and f2 [33]. The f1 index is related to the whole body exposure to γ radiation from K-40 (potassium), Ra-226 (rad), Th-228 (thorium) and should not exceed 1.2. The f2 index denotes the content of Ra-226, thus indirectly Rn-222 and its α radiation, and should be max. 240 Bq/kg. Cement and autoclaved aerated sand concrete have low f1 and f2 indexes [34]. Wooden buildings usually do not have as solid foundations as brick ones, which promotes the diffusion of radon from the ground to the building.

Taking all of the above into consideration, the awareness of the varying radon content in the soil depending on the region could contribute to least partially influencing the radon concentration in houses. The next factor is the selection of building materials, the type of the building and quality of workmanship. It seems more favorable to live in a house with tight concrete foundations. In everyday practice, frequent ventilation of an apartment remains the most important. Unfortunately, it is not always possible due to outdoor air pollution such as smog. Moreover, some energy conservation interventions can influence the indoor radon concentration. An example of such a phenomenon is thermomodernisation of buildings which results in worsening ventilation, leading to an increase in radon concentration. In France, Czech Republic and Russia the concentration of radon after sealing the buildings increased by 1.6 times, and in England, 1.7 times [35]. The US Environmental Protection Agency (EPA) recommends that every house be tested for radon concentration [36].

## 5. Legal Regulations

The level of risk awareness is essential for the expansion of preventive programs. Perception of risk raises concerns and causes individuals to make proactive decisions to mitigate risks. Polish and international law to some extent regulates the exposure to radon.

Baeza et al. in their research work focused on the relationship between the architectural style of single-family houses built between the 18th and 21st centuries and the concentration of radon inside them. The old, traditional houses made mainly of wood and granite; houses from 1940–1980, which were characterized by the presence of concrete, thinner walls and modernized air conditioning systems; as well as new houses, subject to the requirements of construction law, with a predominance of cement and brick and waterproof foundations, were considered. The study showed a higher concentration of indoor radon in new and traditional renovated houses, which is probably related to their increased airtightness, reducing air exchange with the external environment. On the other hand, the current building policy tries to minimize the concentration of radon in new buildings [37]. The Italian National Institute of Health in this year’s report also emphasizes the implementation of legal solutions limiting the concentration of radon in already existing buildings [38]. Such a solution was introduced by the Norwegian authorities in 2009, with the goal of reducing the radon concentration in all buildings. The specific radon prevention measures is usually a radon membrane over the entire base area of the building in combination with a passive radon sump system, activated at indoor radon concentrations above 100 Bq/m^3^. The assumed maximum level of indoor radon was 200 Bq/m^3^ [39].

The United States Environmental Protection Agency advises home buyers to either take radon measurements or verify that the home has been tested for radon, and verify that the home meets the structural conditions that protect against radon exposure. According to the EPA, a homeowner should strive to reduce radon if its concentration is 4 pCi/L or more. In such a situation, it may be helpful to use a fan or a vent pipe. It may be a good idea to hire a specialist (radon-reduction contractor). The EPA places emphasis on controlling radon concentration after every change in the apartment, when changing floors, and carrying out periodic control measurements. The EPA recommends that the water supplied to the home be tested if it comes from a private well and the indoor radon level in the air is increased, as water may be the source of radon in the air. In this case, you can use either point-of-entry or point-of-use filter systems. The target indoor radon concentration should be 2 pCi/L or less [40].

An analysis carried out in Great Britain [21] shows the economic and health benefits of reducing indoor radon concentration below 200 Bq/m^3^, especially in new homes, but also in existing ones.

The European Commission has imposed an obligation on its member states to define geographic areas with a higher risk of high levels of indoor radon (“radon prone areas”) [41].

The Centers for Disease Control and Prevention’s (CDC’s) National Comprehensive Cancer Control Program (NCCCP) emphasizes the role of education and radon control [22].

Polish regulations require periodic testing of drinking water if the concentration of radon activity in water exceeds 10 Bq/L. Exposure is considered to be low between 10 and 100 Bq/L [42]. However, it is not always possible to ensure compliance with such norms, for example, the concentration of radon tested in Jelenia Góra was elevated, which may be associated with the presence of uranium-rich rocks. It was related to estimated exposure to an annual radiation dose of 0.9 mSv from drinking water alone, which corresponds to an average concentration of 200 Bq/L [43]. Potable waters are also tested for radium and tritium [44].

Council Directive 2013/59/EURATOM of 2013 and the Atomic Law Act amended in 2019 specifies the average annual reference level of radon concentration in residential buildings and workplaces, assuming the threshold of 300 Bq/m^3^ [6,45]. Under the Directive, Member States are obliged to plan actions to reduce exposure to radon from soil, water and building materials in buildings, apartments, workplaces and public spaces. In many countries, the norm is 200 Bq/m^3^, although even this amount can double the risk of developing lung cancer [4]. The guidelines of the International Atomic Energy Agency (IAEA) specifies the acceptable average annual radon concentrations for residential and public buildings at 300 Bq/m^3^ and 1000 Bq/m^3^ for workplaces [46]. In public buildings, in special cases, the average annual radon concentration may reach a maximum of 1000 Bq/m^3^ [6].

Also, the ordinance of the Council of Ministers on the limit doses of ionizing radiation specifies the effective dose limits, which should not exceed 1 mSv per year for pregnant women and the general population, and 20 mSv for employees [47].

However, awareness-raising programs are also important. The key point of policies of many countries is the need for a wider coverage of residents with information about the risk of radon exposure and how to reduce it. Efforts to improve public awareness have had some success in some countries [48]. Recent studies show that low-income rural citizens are unaware of the harmful consequences of radon exposure due to lack of access to adequate information [49]. Disagreement also exists between experts and the secular cult over the severity of the risk of radon exposure. In turn, it is easier for public health authorities to encourage testing and remediation when homeowners are convinced that their property and its residents are at increased risk [50]. Radon exposure risk management should include broader prevention actions at population level, which could be expanded to maximize benefits [48].

Within the project “Radon Prevention and Remediation” (2011) of the European Union, the intercomparison of various European public awareness surveys of the risk caused by radon exposure was performed [48,51]. Based on these results, it can be stated that the society in countries with an established national radon strategy possesses better information about radon than in countries without such of strategy. Throughout all the polls it was perceived that radon can be harmful to health, given the harm radon is quite underestimated compared to other risks. In general, it seems that the knowledge of the possibility of taking action to measure and control radon is also positively correlated with the existence of strategies communication of established risks. General remarks from the results of the implementation of the RADPAR project are the following: risk communication increases the level of information and helps to change the behavior of the society; the information about radon must be communicated continuously; possible measures to reduce radon concentrations should be adopted to local conditions and a stakeholder approach should focus on doctors, pharmacists, home inspectors and architects [48,51].

The risk of radon exposure for public health is summarized in much scientific evidence to substantiate political decisions and demonstrate the need for multilevel interventions. The political strategies of the various countries provide effective recommendations of these actions. They include cost-effective measures for the population, which can be promoted by developing a national program of radon exposure control under the auspices of ministries health, and proposals, which can be applied directly to households with different income levels. The key point of these policies is the need for a wider coverage of residents with information on the risk of radon exposure and how to diminishing it. Some more recent evaluations of the control programs of radon exposure concluded that efforts to inform the population has been successful in raising awareness the public and in encouraging the testing of houses at concentrations of radon. However, it is still difficult to convince populations of the importance of radon exposure control and the obligation taking measures to mitigate the consequences of this phenomenon. Public health policy in the field of risk of exposure to radon should take into account the attitude of the government and a resident in solving this problem [48,52,53].

## 6. Application of Radon as a Method of Treatment

Historically, radon was part of a dangerous trend involving the use of radioactivity in medicine. In the United Kingdom, devices for adding radon to drinking water were sold, and radon compresses were used in the arthritis therapy. What is more, radioactive compounds were administered intravenously. That route of administration caused malignant tumors in patients, mainly bones [54]. The era of universal radon availability ended with the accumulation of evidence of its association with miners’ lung cancer. To this today, however, so-called radon waters have been used in spa and wellness treatments. This is due to the coexistence of two conflicting theories about the impact of radiation on human health.

The National Academy of Sciences Biologic Effects of Ionizing Radiation (BEIR VII) report, based on the available scientific evidence, supports the linear no-threshold theory (LNT), indicating a linear relationship between the radon dose and the adverse health effect [55]. Similarly, according to International Commission on Radiological Protection (ICRP), there is no dose of radon so low that it does not pose a risk of lung cancer [35]. Even the smallest dose of radiation has a carcinogenic potential if it hits the DNA of a cell and causes a genetic mutation.

In opposition to LNT, there is the radiation hormesis hypothesis, whose proponents believe that low doses of radiation are useful for health [56]. This theory is based on the validity of the use of radioactive sources in balneology, specifically in healing waters and inhalations. Radon water is categorized as specific water because it has a specific component, in this case, radon, whose concentration should not be lower than 74 Bq/L [57,58]. Some Polish wellness towns which use radon water include Jedlina Zdrój, Lądek Zdrój, Przerzeczyn, Szczawno Zdrój, Świeradów Zdrój, Długopole Zdrój and Duszniki Zdrój [59]. Radon baths are recommended by the Polish national consultant on balneology and physiotherapy [60].

Radon waters appear to be analgesic and anti-inflammatory and regulate the activity of the autonomic nervous system. Radon probably influences the neuroendocrine system, affecting the adrenal glands via the pituitary gland. Subsequent hormonal changes modulate T-cell function. Radon water therapy is recognized primarily by dermatologists and rheumatologists. Treatments are offered for rheumatoid arthritis, scleroderma, fibromyalgia, infectious joint diseases, neuralgia, chronic post-traumatic pain, potency disorders, menopause symptoms, endocrine disorders, sinusitis and peripheral vascular diseases. They are also recommended in the treatment of allergic diseases, such as atopic dermatitis and allergic rhinitis, as well as in lung diseases—asthma and chronic bronchitis [57]. Research indicates the effect of radon on Langerhans cells, an increase in enkephalin levels and a reduction in the amount of free oxygen radicals in phagocytes [61].

Treatments using radon media increase the synthesis of adrenal cortex hormones, as well as female and male sex hormones. Radon waters have a positive effect on the carbohydrate metabolism, increase the production of B and C vitamins. Radon also has a positive effect on the skin as an anti-inflammatory, antipruritic and analgesic, and also accelerates the regeneration process of the epidermis [62]. Radon treatments improve blood circulation and skin elasticity [63]. A one-time fifteen-minute radon bath increases blood flow through the tissues by about 400%. This congestion lasts over 60 min from the end of the bath. Treatment with radial waters has a positive effect on the parameters of lipid metabolism [62]. Radon also accelerates the regeneration of damaged nerve fibers. The likely mechanism of this action is to cause local overheating and intensify the synthesis and secretion of neurohormones. Radon therapy is also effective in the treatment of peripheral vascular diseases, however, it is recommended to use waters with high radon content, such as in the waters of Lądek Zdrój or Świeradów Zdrój in Poland [64].

Kojima et al. provided case reports of two patients with pemphigus and type I diabetes who benefited significantly from radiotherapy that employed low doses of nontargeted α-radiation and its associated β- and γ-radiations, delivered by the inhalation of radon (in a typical radon spa). Radon therapy, properly delivered, has been shown to induce important remedies that provide relief from severe suffering and the possibility of return to normal living. Moreover, the authors did not observe any side effects after this therapy [65].

Due to exposure to low doses of radiation and the potential risk of induction of carcinogenesis, from the very beginning of treatment with radon media, the question of their dosage is the most controversial. The first problem is the daily and seasonal variability of the radioactivity of natural radon sources, which decreases both during long-term precipitation and in its absence and increases with increasing atmospheric pressure. Often, the measurements of the activity of radial waters are limited to the determination of the radial activity of the healing water source itself, ignoring the changes that occur in the water during the preparation of baths or pools. Heating the radon water during the preparation of the bath reduces the solubility of radon, which depends on temperature and pressure. Heating the radon water causes an average loss of radon content of 50–70% [62].

The legitimacy of using the potentially dangerous treatment methods in the light of the availability of many other therapies with proven effectiveness remains debatable. The potential risk/benefit ratio should be carefully assessed due to the strong evidence indicating the health-threatening effects of radiation emitted by radon.

## 7. Impact of High Doses of Radiation on Human Health

Natural sources such as radon emit small doses of radiation. Therefore, their negative health effects do not manifest as the classic radiation sickness caused by contact with man-made high-energy radiation. Such radiation would be emitted as a result of an atomic bomb explosion or a nuclear power plant disaster. In nearly three-quarters of century since the Hiroshima and Nagasaki bombings and a quarter of a century since the explosion at the Chernobyl nuclear power plant, many studies have been carried out on the effects of high doses of radiation on living organisms [66,67,68,69,70].

Depending on the intensity of the absorbed radiation, the area of the body exposed to the radiation and the duration of exposure, the biological effects will vary. Unfortunately, in extreme situations, they can lead to immediate death. The survivors may develop various types of complications, starting from the molecular level and ending with the systemic one.

Acute radiation syndrome can occur in a person after when the dose is at least one gray (Gy), or one joule of energy per kilogram of body weight. The most sensitive element of the human body are mature lymphocytes, the number of which decreases as soon as one day after exposure. Subsequently, bone marrow is damaged. At a dose of about 5 Gy, the disease affects other organs, in particular the digestive system, followed by the skin and central nervous system. The prodromal symptoms of the acute illness include nausea, vomiting, diarrhea and abdominal pain, which can be caused by transient activation of the autonomic system. Chronic radiation sickness can develop even at doses lower than 1 Gy. It affects similar organs as the acute disease [7,71]. An additional long-term effect of radiation exposure is a malignant tumor. This phenomenon can occur even as a result of exposure to elevated concentrations of radon originating from natural sources.

## 8. Radon Is an Aetiological Factor in Cancer and Noncancerous Diseases

### 8.1. Cancer Diseases

Radon is considered a carcinogenic agent [72]. Carcinogenesis is a multifactorial process leading to the formation of cancer. It is a long-term process that disturbs the balance between proliferation, apoptosis, differentiation and aging of cells, and its course depends on the type of tumor and the tissue in which it occurs [73]. The causes of the carcinogenesis process are hereditary and spontaneous mutations induced by chemical and physical factors. They concern genes responsible for the control of the life cycle: suppressor genes, proto-oncogenes and regulatory genes. Changes in the nucleotide sequence in the DNA chain result in uncontrolled fragmentation of the mutated cell, which leads to neoplastic transformation [74]. In studies that were carried out on an animal model, it was found that there are three basic stages in the process of carcinogenesis: initiation, promotion and progression. In the initiation stage, an irreversible change of a genotypic nature occurs, which consists in DNA damage caused by the interaction with a reactive form of a carcinogen [74]. The second phase of carcinogenesis is promotion, which is the result of the incorporation of promoting carcinogens (promoters). At the stage of promotion, epigenetic changes take place, and there is a selective clonal growth of the initiated cells by increasing proliferation or inhibiting apoptosis. As a result, phenotypic changes occur, preneoplastic damage to the mutant cell occurs, and specific functions are lost and connectivity with other cells occurs. The last stage of carcinogenesis is progression, which is an irreversible stage, including invasion of adjacent tissues and metastasis to distant organs [75]. A carcinogen is a mutagen that causes DNA damage. Depending on the nature and way of action of carcinogens, they can be divided into two groups: genotoxic and epigenetic. Genotoxic carcinogens, which bind to DNA, initiate and cause the progression of mutations necessary for tumor development. Any exposure to genotoxic carcinogens may carry a risk of inducing cancer as they are nonthreshold factors. This means that it is not possible to define a safe concentration (threshold) that does not cause any changes in the body. The genotoxic carcinogen class includes direct factors that do not require metabolic activation, and indirect factors (carcinogens) whose metabolites are direct carcinogens. Epigenetic carcinogens, which do not bind to DNA, activate proto-oncogenes as a result of disturbed signaling pathways and accelerate the process of carcinogenesis by promotion or immunosuppression [76].

The pathogenic effect of radon (and above all its decay products remaining in the respiratory system) is associated with the emission of ionizing radiation. Such radiation may, directly and indirectly, damage the genetic material contained in the cell nucleus DNA. It breaks the DNA double-strand [77]. The indirect effects are based on water radiolysis and the generation of reactive oxygen species. DNA damage causes mutations leading to cell carcinogenesis, resulting in the development of tumors. The European Code Against Cancer announced twelve principles for cancer prevention in 2015. One recommendation was to reduce exposure to high levels of radon [78]. In Europe, it is estimated that radon present in houses is responsible for 2% of deaths from malignant neoplasms [79]. The correlation of radon with the incidence of lung cancer has been proven beyond doubt, however, that this element can probably also cause kidney cancer, melanoma, as well as haematological cancers and primary brain tumors [80,81]. The relationship between radon and cancer of the stomach, liver and pancreas is unresolved [82], while no such correlation has been proven for throat and oral cancer [83].

Radon has little potential for penetration into systems other than the respiratory system, and therefore its association with diseases other than respiratory diseases is uncertain. There is still no scientific evidence to relation of radon to diseases other than lung cancer. The available data are conflicting even in the best-studied diseases such as leukemia. WHO suggests treating the results of such research with caution as it may be misleading [23]. Moreover, even finding a significant statistical correlation does not explain the mechanism of carcinogenesis. Taking into account the kinetics of radon, its place of action should be primarily the bronchi and lungs, and in the case of drinking water with increased content of the gas, the digestive system.

#### 8.1.1. Occupational Radon Exposure

It is estimated that millions of people are currently working underground, and their exposure to radon is significantly higher because it is not diluted in the atmosphere. It is worth remembering that its concentration underground can be reduced through ventilation systems and radon-proof barriers [84]. Most of the research on the effects of radon on human health was carried out on miners who were exposed to higher doses of radon in their work environment than ordinary people in their place of residence. Pooled data from eleven studies conducted on miners [85] did not show a significant association between cumulative radon exposure and mortality from nonlung cancer. While there are studies showing such correlations, miners’ exposure to numerous environmental factors other than radon does not reliably link radon exposure to lung diseases other than lung cancer [86].

A statistically significant correlation was found in miners with a greater incidence of leukemia, especially chronic lymphocytic leukemia (CLL), while a positive correlation was not significant in the case of Hodgkin’s lymphoma and myeloid leukemia considered separately [87]. Colorado miners showed a higher incidence of non-Hodgkin’s lymphoma and multiple myeloma [82]. However, another study [88] conducted on a population of miners did not confirm the relationship between radon and leukemia, but showed a significant increase in mortality associated with cancer of the liver, gallbladder and extrahepatic bile ducts, but it was not possible to demonstrate a causal relationship between exposure to radon and the occurrence of these events. Similarly, a higher incidence of liver and stomach cancer was observed in miners from Kazakhstan’s uranium mines [53]. Morrison et al. found a relationship between radon and oropharyngeal cancer, but the number of cases detected was too small to be reliable [89]. In the case of miners examined in Silesia, an increase in the incidence of lung and larynx cancer was noted, and miners who came to work from another region suffered more often [90]. The relationship between radon and central nervous system (CNS) neoplasms observed in miners remains uncertain [91].

#### 8.1.2. Indoor Radon Exposure

Initially, the results of research conducted on miners were extrapolated to other populations, which was associated with a high risk of error. The first large studies attempting to show the relationship between radon in the living environment and cancer were based mainly on average radon concentrations obtained from databases, and not measured by researchers, which limited the credibility of these studies [92]. Other environmental studies have provided inconsistent and often statistically insignificant data on the relationship of radon to hematological malignancies [93,94]. Many other environmental studies have been dedicated to leukemia, especially among children, but the results are contradictory.

Some ecological studies have shown a relationship between mean radon levels and childhood leukemia incidence and mortality rates [95,96,97,98]. Recently, a newly prediction model was used in a large nationwide Danish study to calculate indoor radon levels in the homes of children with cancer and control children. The authors found a significant relationship between cumulated radon exposure and risk for acute lymphoblastic leukemia in children [95]. Also, dosimetric calculations done in the UK have indicated that about 6% of childhood leukemia’s might be due to domestic radon [99]. Statistical associations of radon with thyroid, skin and kidney cancer have been observed, however, other overlapping causes of these diseases cannot be excluded, and therefore the relationship remains uncertain [91].

Some studies have been done on the association between skin cancer and radon. The American Cancer Prevention Study II cohort noticed hazard ratios of 1.08 (95% CI: 0.88, 1.33) and 0.70 (95% CI: 0.42, 1.19) per 100 Bq = m^3^ in mean county-level residential radon for malignant melanoma and nonmelanoma skin cancer mortality, respectively [100]. Wheeler and co-authors revealed an association between radon and occurrence of squamous cell carcinoma, but not basal cell carcinoma or malignant melanoma, in southwest England. Moreover, they didn’t found an association with occurrence of nonmelanoma skin cancer at the national level [101]. In another study, the authors found a statistically significant association between basal cell carcinoma and radon, but not for malignant melanoma or squamous cell carcinoma in a Danish cohort research [102]. Vienneau et al. found an increased risk of skin cancer mortality in association with household radon levels in Switzerland. Their study supports the hypothesis that radon exposure is a relevant risk factor for skin cancer independent of residential erythemal-weighted radiation exposure. The authors found a statistically significant increased risk of death from malignant melanoma and skin cancer in general, independent of erythemal-weighted radiation, in adults associated with exposure to radon. Switzerland has amongst the highest skin cancer incidence that may be related to the wealth and behavior of the population leading to recreational UV radiation exposure. In addition, natural UV levels are also relatively high owing to the elevation in the alpine regions. Moreover, certain areas of Switzerland have elevated radon levels because of the underlying geology, which leads to high doses of radon [100].

### 8.2. Noncancerous Diseases

Radon can also cause noncancerous diseases and affect their course. A relationship is observed between radon exposure and chronic obstructive pulmonary disease (COPD) development and the frequency of hospitalizations, especially in women [103]. Miners exposed to radon were more likely to die from silicosis and pulmonary fibrosis, but also end-stage renal disease in diabetic nephropathy [82]. Schubauer-Berigan and coauthors found that cumulative silica exposure is related to cumulative radon exposure, because they are correlated with duration of mine employment. The authors observed a strong gradient between silicosis standardized mortality ratios and radon exposure. This finding suggests that miners with high radon exposures also had raised silica exposures, likely during early time periods when silica exposures were not controlled [47]. Alternatively, radon-related pulmonary diseases may have been misattributed as silicosis, leading to elevations in the silicosis standardized mortality ratios for the higher working level month (WLM) groups. A relationship between exposure to higher radon concentrations and the occurrence of congenital malformations such as cleft lip and palate and cystic lymphangioma has also been shown [104]. Correlation with radon exposure was also sought in patients with neurodegenerative diseases, in particular multiple sclerosis, however, studies by Groves-Kirkby et al. showed no statistically significant relationship [105].

Research conducted on the population living in the region of Kazakhstan, rich in uranium mines, showed a greater number of women with impaired fertility and children with urinary tract diseases and chronic bronchitis [53]. Seo et al. also studied a relation between radon exposure and heart disease, but research results are uncertain [91]. Blomberg and coauthors observed higher mortality due to cardiovascular and respiratory diseases with the simultaneous occurrence of increased concentrations of PM2.5 and radon. The authors indicate that PM2.5 can act as a vector for radon, transporting it to the bronchial tree [25].

It should be remembered that water is an important source of radon. Radon can pass from water to air, and in the case of drinking water, it can also reach the digestive tract. The National Research Council estimates that approximately 30% of the radon-222 reaching the stomach enters the stomach wall, suggesting its potential to cause stomach cancer [106]. Several studies have been conducted between the concentration of radon in drinking water and the occurrence of gastrointestinal malignancies, but their results are contradictory [107,108].

## 9. Radon and Lung Cancer

The special relationship of radon with lung cancer, as well as with other pulmonary diseases, results from its physicochemical properties. As a gas contained in atmospheric air, it can easily be inhaled into the bronchial tree, where it is deposited especially in bronchial bifurcations [109]. Its labile radioactive derivatives, i.e., polonium, bismuth and lead (Po-218, Pb-214, Bi-214, Po-214), reach the lungs. Po-218, which deposits in the bronchial tree and emits α and β radiation, is particularly dangerous [4]. α-radiation has a short-range and can be easily shielded, but in the lungs, it has direct access to epithelial cells and can damage genetic material. The dose dependence of radon carcinogenic potential has been demonstrated, and a marked increase in lung cancer risk has been observed at long-term exposure to 100 Bq/m^3^ [23,110].

Vahakangas and coauthors detected mutations in the p53 protein gene in miners exposed to high doses of radon [111]. The p53 protein is coded by tumor suppressor gene TP53 localized in on chromosome 17p13.1 [112]. The p53 protein gene is the site of the most common inactivating mutations in patients with lung cancer [4]. Experimental studies of Liu et al., involving high-dose radon irradiation of bronchial epithelium, showed its effect on p53-related metabolism, particularly apoptosis and glycolysis [113]. In studies conducted by the same author on miners from a tin mine, attention was also paid to the synergistic effect of radon with arsenic in inducing bronchial epithelial mutations [114]. Experiments performed by Chen et al. on rats and human bronchial epithelial cells showed an increase in the production of reactive oxygen species, down-regulation of let-7 microRNA and an increase in the expression of the KRAS oncogene [4,115]. Similarly, no differences were found in the frequency of EGFR gene mutations, while ALK rearrangement was slightly more frequent at higher radon concentrations [116]. Taga et al. raised the hypothesis that the *EGFR* mutations can be related to residential radon or second-hand smoking. Although they observed a high frequency of *EGFR* mutations (41%) in lung tumors in never smokers or long-term former smokers, but no association between *EGFR* mutations with neither secondhand smoking nor exposure to residential radon was found [117]. On a large group of patients with non-small-cell lung cancer, Mezequita investigated the relationship between the intensity of radon exposure and the occurrence of specific types of mutations such as EGFR, BRAF, KRAS and HER2, as well as ALK and ROS1 rearrangements. It was observed that the occurrence of EGFR, BRAF, HER2 and ROS1 was significantly higher in areas with high radon exposure, while KRAS mutations were more frequent in areas with low exposure [118]. More recent research of Mezquita et al. tried to answer the question if there is any link between the genetic diversity of non-small-cell lung cancer and exposure to indoor radon, Although it was conducted in a small group of patients the research led to the conclusion that indoor radon concentrations exceeded those recommended by WHO, and despite the fact that there were no differences between groups with EGFR, ALK, and BRAF patients, the concentrations above the WHO recommendations were most common with ALK rearrangement and BRAF mutation [119].

It should be noted that EGFR mutations and ALK rearrangements offer the possibility of targeted therapy in patients with inoperable lung cancer [120]. Miners exposed to radon showed elevated levels of interleukin 6, which is secreted by pulmonary fibroblasts and may contribute to both cancer and COPD [121,122].

The main aetiological factor in lung cancer is smoking, which can account for up to 90% of deaths from this cancer. Lung cancer patients are often stigmatized due to the indisputable effect of smoking on lung cancer [123]. It should be emphasized, however, that lung cancer also occurs in people who have never smoked.

Work-related factors account for 9–15% of deaths and radon accounts for 10%, while the impact of air pollution is at a level of 1–2% [4]. The synergistic effect between smoking and radon is indisputable. As many as 86% of deaths from lung cancer associated with radon exposure occurred in smokers and former smokers [124]. Therefore, it seems that actions taken to discourage smoking would be more beneficial than just lowering radon levels in the human environment. However, it should not be forgotten that radon remains the second independent lung cancer risk factor after smoking, and therefore the first aetiological factor in the population of never smokers [4,23,121].

In the US, 15–21 thousand deaths a year from lung cancer are associated with radon [4,124]. Radon is associated with all histological types of lung cancer. It most frequently causes adenocarcinoma. It also contributes to squamous cell carcinoma [122,125]. It has been shown that miners in uranium (radon precursor) mines die even three times more often from lung cancer [4]. An analysis of eleven studies carried out on miners showed an increased risk of death from lung cancer per working level month (WLM), and the relationship between this risk and the cumulative dose was almost linear [23]. In one study, radon was responsible for 40% of deaths from lung cancer among miners, including 39% in smokers and 70% in never-smokers. For the sake of comparison, the value for houses was 10% of deaths, including 11% in smokers and 30% in never-smokers [126]. In the UK, 1100 deaths are reported annually from lung cancer related to exposure to indoor radon [21]. Research has shown that there is a clear relation between radon in concentrations typical for residential buildings and the risk of developing lung cancer [23].

Some studies show an additive or even hyperadditive effect of simultaneous smoking and radon exposure on the risk of lung cancer. It has been shown that, with the same radon exposure, smokers have a greater risk of developing lung cancer than nonsmokers [127]. It has been suggested that this effect occurs at high radon concentrations. A greater risk of lung cancer associated with radon exposure has been shown in nonsmokers, but the absolute risk is greater in smokers due to the predominant influence of cigarettes on carcinogenesis [92,128].

The research conducted on rats comparing the synergic effects of exposure on radon and other airborne pollutants, for example, tobacco smoke, beta-naphthoflavone, mineral fibers and diesel exhausts. The strongest link was shown between the combined effect of radon and tobacco smoke and the incidence of lung cancer. This effect decreased as exposure to tobacco smoke was limited [129].

A similar effect was also shown in humans. In the matched case-control study conducted in Korea it was proved that exposure to residential radon and smoking had a synergistic effect increasing the risk of developing lung cancer [130].

## 10. Conclusions

In the light of modern knowledge, the relationship between lung cancer and radon exposure remains undeniable. It is known that radon is the second most common cause of lung cancer in smokers and the most common one in nonsmokers. Even though WHO deemed radon exposure as carcinogenic, knowledge about this threat remains incomplete and superficial. The need to inform the medical professionals and the public about the carcinogenic effect of radon exposure has been indicated by the work of American authors who conducted a systemic review of 20 studies on public understanding of the impact of radon exposure on lung cancer development [131]. It has been shown that even in a group of people confirming that they have heard of radon, only slightly more than 20% are aware of the relationship between radon and lung cancer. Even in the group of people who conducted radon tests at home, only 50% have such understanding. This work also drew attention to the problem of misunderstanding the issue of radon exposure and mistaking the symptoms associated with radon exposure for symptoms corresponding to carbon monoxide poisoning.

Our article is part of the process of popularizing the issue of radon exposure and its carcinogenic effect.
